# Neurocognitive and neuroinflammatory correlates of PDYN and OPRK1 mRNA expression in the anterior cingulate in postmortem brain of HIV-infected subjects

**DOI:** 10.1186/1742-2094-11-5

**Published:** 2014-01-09

**Authors:** Vadim Yuferov, Eduardo R Butelman, Ann Ho, Susan Morgello, Mary Jeanne Kreek

**Affiliations:** 1 Laboratory of the Biology of Addictive Diseases, The Rockefeller University, 1230 York Avenue, New York, NY 10065, USA; 2 Department of Neurology, Neuroscience and Pathology, Mount Sinai Medical Center, Annenberg Building Room 14-66, 1 Gustave Levi Place, Box 1137, New York, NY, USA

## Abstract

Chronic inflammation may contribute to neuropsychological impairments in individuals with HIV, and modulation of this inflammatory response by opiate receptor ligands is important in light of the prevalence of drug use in HIV populations. Exogenous MOR and KOR agonists have differential effects on central nervous system (CNS) immunity and, while some data suggest KOR agonists are immunosuppressive, the KOR agonist dynorphin has been shown to stimulate human monocyte chemotaxis. In this study, we examined mRNA levels of endogenous opioid receptors *OPRK1* and *OPRM1*, prodynorphin (*PDYN*), macrophage scavenger receptor CD163, and microglia/macrophage marker CD68 in the caudate and anterior cingulate of postmortem brains from HIV-positive and HIV-negative subjects. Brain tissues of HIV-infected (n = 24) and control subjects (n = 15) were obtained from the Manhattan HIV Brain Bank. Quantification of the gene mRNA was performed using SYBR Green RT-PCR. CD68 and CD163 were increased in HIV-positive (HIV+) compared to HIV-negative (HIV-) individuals in both brain regions. There were higher *OPRK1* (*P* <0.005), and lower *PDYN* mRNA (*P* <0.005) levels in the anterior cingulate of HIV+ compared to HIV- subjects. This difference between the clinical groups was not found in the caudate. There was no difference in the levels of *OPRM1* mRNA between HIV+ and HIV- subjects. Using linear regression analysis, we examined the relationship of *OPRK1* and *PDYN* mRNA levels in the HIV+ subjects with seven cognitive domain T scores of a neuropsychological test battery. Within the HIV+ subjects, there was a positive correlation between anterior cingulate *PDYN* mRNA levels and better T-scores in the motor domain. Within the HIV+ subjects there were also positive correlations of both *OPRK1* and *PDYN* mRNA levels with the anti-inflammatory marker CD163, but not with proinflammatory CD68 levels. In this setting, decreased *PDYN* mRNA may reflect a homeostatic mechanism to reduce monocyte migration, accompanied by compensatory increases in the cognate receptor (KOR) to dampen pro-inflammatory responses. It is possible that enhanced neuroprotection and better motor performance are associated with higher levels of dynorphin and the recruitment of neuroprotective CD163-positive macrophages. Further studies are needed to test this hypothesis.

## Background

Despite the availability of combination antiretroviral therapy (CART), which has successfully controlled HIV viremia and improved immune function in many treated HIV-infected patients, HIV-associated neurocognitive disorder (HAND) remains highly prevalent [1]. The pathogenesis of HAND is still unclear, and is very often associated with nonviral neurobiological factors [2,3]. Numerous studies suggest that HAND is primarily the result of neuronal loss/dysfunction from direct or indirect viral effects, inclusive of inflammation driven by chronic low-level infection, loss of trophic factors, and elaboration of excitotoxic molecules (for example, [4]). Morphologically, HIV-associated cognitive impairment has been linked to alterations in the synaptodendritic network in HIV-infected brain [5,6]. Currently, it is commonly accepted that cytokines and chemokines secreted by activated microglia and astrocytes in inflammatory conditions lead to alterations in synapse and dendritic spine structures in HIV-infected subjects, with a major role ascribed to glutamate neurotoxicity.

Despite numerous reports of effects of exogenous opiates, particularly drugs of abuse, on replication of HIV, HIV-associated neurotoxicity and modulation of immune responses in cell culture, animal models, and AIDS pathology in humans, (for example, [7,8]) much less is known of the impact of the endogenous opioid system on HIV neuropathogenesis and HIV-associated neurocognitive impairment. The opioid system comprises the mu (MOR), delta (DOR) and kappa (KOR) opioid receptors, which are activated by the endogenous opioid peptides beta-endorphin, enkephalin and dynorphin, respectively (for example, [9] for review). *In vivo* and *in vitro* studies showed that stimulation of opioid receptors by exogenous MOP agonists like morphine leads to suppression of multiple components of the immune response including phagocytosis, natural killer cell activity, chemokine-induced chemotaxis, antibody response and cell-mediated immunity ( for review [10]). Several reports demonstrate that Dynorphin A modulates the capacity of immunocytes to enhance or suppress chemotaxis through direct or indirect stimulation of KOR. Ruff *et al. *[11] have shown that Dynorphin 1-13 is a potent stimulator of human mononuclear cell chemotaxis. In recent studies of bi-directional heterologous desensitization between the chemokine receptor CXCR4 and KOR, Finley *et al*. [12] showed that treatment of the Jurkat T cell expressing KOR and CXCR4 with the KOR agonist U50,488H diminished the chemotaxis response to chemokine CXCL12. In the context of HIV, chronic opiate exposure has been associated with decreased expression of macrophage activation markers in brain [13]. In contrast to mu opioid receptor ligands, dynorphin peptides (primarily endogenous KOR agonists) decrease basal and drug-induced dopamine levels in several areas of the dopaminergic nigrostriatal and mesolimbic-mesocortical systems as well as in tuberoinfundibular dopaminergic (TIDA) neurons in the hypothalamus [14]. In animal models, dynorphin/KOR system activation is also implicated in depression and anxiety, which may be secondary to the dopaminergic modulation [15]. In humans and nonhuman primates, exogenous high-efficacy κ-opioid-receptor agonists have dose-dependent central nervous system (CNS)-mediated effects that include sedation (for example, unresponsiveness to environmental stimuli), dysphoria, anhedonia, depressive symptoms and psychotomimesis [16-18].

In different experimental models of neurodegeneration and traumatic brain injury (TBI), dynorphin was shown to be either neuroprotective [19-21] or neurotoxic [22]. In the context of HIV infection, kappa opioid receptor ligands have demonstrated potential anti-inflammatory and neuroprotective properties in several *in vitro* models of HIV neuropathogenesis. The synthetic KOR agonist (for example, U50,488) suppresses HIV-1 production in human microglial cells [23] and CD4 T lymphocytes [24], and dampens chemokine production in astrocytes [25]. However, it has been shown that dynorphin stimulates TNF-a and IL-6 expression in human brain cell cultures, and the stimulatory effect of dynorphin resulted in upregulation of HIV-1 expression when human brain cells were co-cultured with human promonocytic cells U1 [26].

In the present study we have examined expression of opioid genes *OPRM1*, *OPRK1* and *PDYN* in two brain regions, the caudate (a terminal field of the dopaminergic nigrostriatal system) and anterior cingulate (a terminal field in the mesocortical dopaminergic system) of postmortem brain of HIV-infected and control subjects; these areas are known to contain opioid receptors in humans [27]. Several studies suggest that HIV-mediated neuropathogenesis includes the loss of dopaminergic terminals in the basal ganglia, including the caudate and putamen, either through degeneration of dopaminergic neurons in the substantia nigra or via local HIV-induced striatal pathology. This is postulated to lead to deficits in central dopaminergic activity, resulting in progressive impairment of diverse neurocognitive and motor functions [28,29]. The anterior cingulate cortex (ACC) is a heterogeneous subregion of the prefrontal cortex. Functions of the ACC include cognitive and attentional processing, autonomic regulation, motor control, and emotional control. [30]. Studies on the distribution of cortical dopamine neurons in primates showed that the dopamine innervation is most dense in the motor and anterior cingulate cortex [31]. Recently, decreased levels of the preproenkephalin mRNA (*PENK*) and dopamine receptor D2 (*DRD2*) in the dorsolateral prefrontal cortex (DLFPC) in postmortem brain of subjects with HIV/AIDS has been reported [32].

In order to investigate whether there is an impact of the KOR/PDYN system and *OPRM1* on HIV-related neuropsychological impairment, we examined the postmortem brains of HIV-infected and control subjects to identify any changes in quantitatively measured levels of *PDYN*, *OPRK1* and *OPRM1* as well levels of macrophage markers CD68 and CD163 in the caudate and anterior cingulate. We have found lower *PDYN* and greater *OPRK1* mRNA levels in the anterior cingulate in HIV+ subjects. There was a positive correlation *PDYN* and *OPRK1* levels with expression of macrophage/microglia marker CD163 in the anterior cingulate of HIV+ subjects. Furthermore, there was a positive correlation between better T-scores in motor domain scale and *PDYN* mRNA levels in this region.

## Methods

### Study participants

A description of the 24 HIV+ and 15 HIV-seronegative unrelated subjects from whom postmortem brain samples were obtained has been reported recently [33], and information for HIV+ subjects is presented in Additional file 1: Table S1. In brief, brain tissues were obtained from the Manhattan HIV Brain Bank, a member of the National NeuroAIDS Tissue Consortium (MHBB, The Mount Sinai Medical Center, New York, NY, U24MH100931). The MHBB operates under local IRB-approved ethical guidelines, and written informed consent was obtained from all subjects, or their primary next-of-kin, for collection and use of autopsy tissues for medical research and furthering medical knowledge. Specimens from subjects with protracted agonal state, as manifested by extensive anoxic-ischemic damage on histological evaluation, were excluded from this study. Mean ages (years ± SD) were 52 ± 10 in HIV-seronegative and 45 ± 10 in HIV-seropositive subjects, and corresponding postmortem intervals (hours, PMI) were 18.4 ± 6.2 and 9.3 ± 5.0, respectively.

### Neurocognitive test assessment

A neuropsychological battery of tests was used to assess the following seven cognitive domains, as previously described: verbal fluency, attention and working memory, executive functioning, learning/memory encoding, memory retrieval, information processing speed, and motor ability [34]. Raw scores from all tests were converted to demographically adjusted T-scores that adjusted performance for effects of age, education, sex and ethnicity. T-scores for each test were averaged to yield domain T-scores for each cognitive domain [see Additional file 1: Table S1]. T-scores are normally distributed and have a mean of 50 and a standard deviation of 10; T-scores more than one standard deviation below normative (<40) were considered impaired. HIV-negative subjects were chosen on the basis of normal premortem neurological function and normal postmortem brain histology.

### RNA preparation and cDNA synthesis

RNA extraction from the caudate and anterior cingulate was performed as described previously [31]. In brief, brain tissues were homogenized in RLT buffer (RNeasy Mini Kit, Qiagen, Valencia, CA, USA) for isolation of total RNA according to the manufacturer’s protocol. RNA samples were treated with RNase-Free DNase (TURBO DNA-free, Ambion, Austin, Texas, USA). RNA preparations were analyzed using an Agilent 2100 Bioanalyzer (Agilent Technologies, Santa Clara, CA, USA). Mean RNA Integrity Number (RIN) values (± SD) were 7.4 ± 0.96 (range from 5.3 to 9.2) in HIV-seronegative and 7.3 ± 1.5 (range from 5.0 to 9.5) in HIV-seropositive subjects. Single-stranded cDNA was synthesized using approximately 1 μg of RNA and the High Capacity cDNA Reverse Transcription Kit (Applied Biosystems (ABI), Carlsbad, CA, USA) in the presence of random primers and gene-specific antisense primer.

### Quantitative real-time PCR

Quantification of the mRNA levels of opioid genes *OPRK1*, *OPRM1*, *PDYN*, and macrophage/microglial markers CD68 and CD163 in the caudate and anterior cingulate cortex, was performed by real-time polymerase chain reaction (qRT-PCR). cDNA (2 μl) was amplified in a 20-μl solution that contained the Brilliant III Ultra-Fast SYBR™ Green QPCR Master Mix (Agilent Technologies) and 10 nM of primers with a PCR condition of 40 cycles of denaturation at 94°C for 5 sec, and annealing/extension at 60°C for 15 sec. Forward and reverse primers for amplification of cDNA of genes studied were ether custom designed or commercially available (SABiosciences, Valencia, CA, USA) [see Additional file 2: Table S2]. Levels of the human glyceraldehyde-3-phosphate dehydrogenase (GAPDH) cDNA/mRNA were used for normalization of levels of mRNA of the target genes. Experimental samples were amplified simultaneously with samples that contained serial dilutions of a target gene and GAPDH cDNAs from 10^1^ to 10^6^ copies/2 μl in sterile water, used to prepare standard curves. Copy number determination was calculated as described previously [33,35]. The qRT-PCR analysis was performed using SDS 2.2 software (ABI) on an ABI Prism® 7900 sequence detection system. The specificity of amplification was confirmed by agarose gel electrophoresis of PCR products, a melting curve profile, and, in some cases, by Sanger sequencing. Copy number of cDNA of opioid receptors, *PDYN*, glial/macrophage markers and *GAPDH* was quantified by comparing threshold cycles (Ct) of an experimental sample to those in standard curves for specific genes and *GAPDH* cDNA. The cDNA copy number is expressed as normalized to copies of GAPDH cDNA copy number.

### Statistical analysis

Normalized values of copy numbers of mRNA of each gene studied were quantified as the natural log of ratio of copy number of gene of interest to copy number of GAPDH cDNA in the caudate and in the anterior cingulate. For expression of each gene in each region, a t-test was used to determine the statistical significance of differences between HIV- and HIV+ subjects, and cognitively impaired and non-impaired HIV+ subjects. The relationship of expression of selected genes with opioid genes and macrophage mRNA levels was examined using Pearson correlation analysis. Correlation analysis was also used to examine whether there was a relationship between expression levels of opioid genes and cognitive status (domain T-scores).

## Results

### Expression of opioid receptor genes

**Figure 1:**
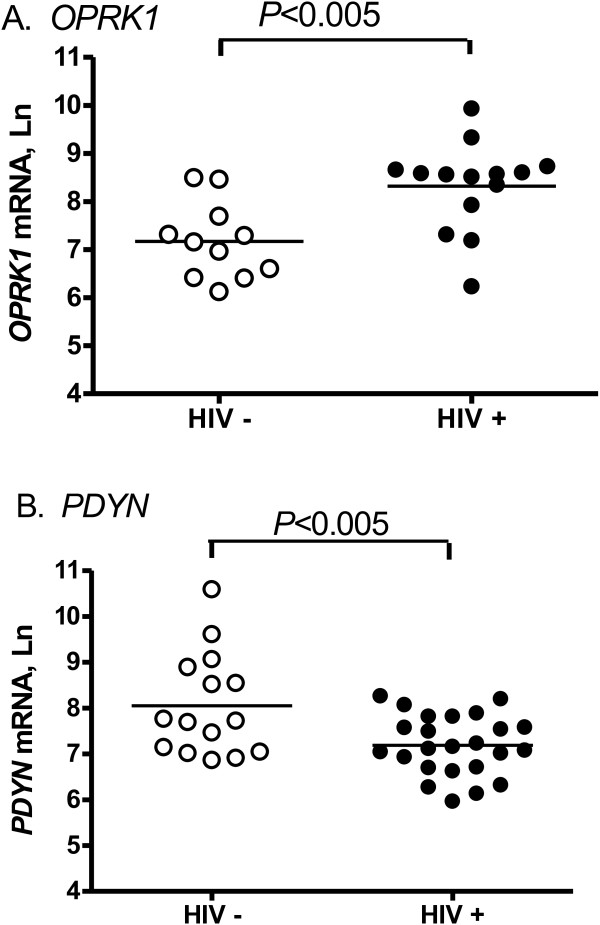
**There are significant differences in mRNA levels of *OPRK1 *(A) and PDYN (B) between HIV-negative and HIV-infected individuals in the anterior cingulate.***PDYN* mRNA levels were lower while *OPRK1* were higher in HIV positive subjects.

**Figure 2:**
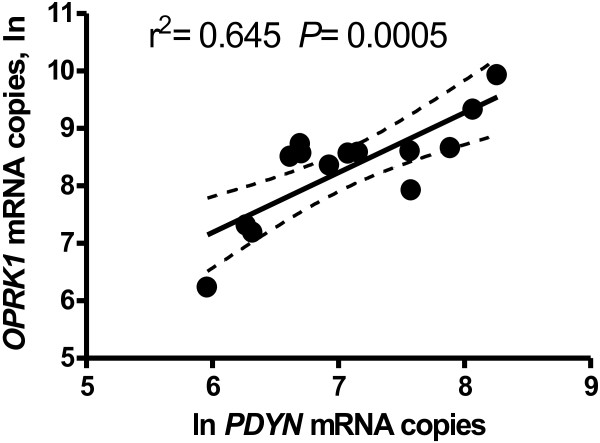
***OPRK1 *** **mRNA levels were significantly correlated with *PDYN *** **in the anterior cingulate of HIV infected subjects.**

We have found significantly higher levels of *OPRK1* mRNA in the anterior cingulate (*P* <0.005) but not in the caudate of HIV+ subjects compared to HIV- subjects (Table 1 and Figure 1A). In contrast, there were significantly lower levels of *PDYN* mRNA in the anterior cingulate of HIV+ subjects (*P* <0.005, Figure 1B), and no difference in *PDYN* mRNA levels between HIV+ and HIV- subjects in the caudate. We did not find a difference in the levels of *OPRM1* mRNA between HIV+ and HIV- subjects in either brain region (Table 1). There was a significant positive correlation of *OPRK1* with PDYN in the anterior cingulate (*P* <0.005) of HIV-infected subjects only (Figure 2).

**Table 1:** The mRNA levels of opioid genes and macrophage/microglia markers in two brain regions in HIV- and HIV+ subjects

	**Caudate**					**Anterior cingulate**				
**Gene**	**HIV-**	**HIV+**				**HIV-**	**HIV+**			
	**Mean ± SEM**	**Mean ± SEM**	***t***	**df**	***P *** **value**	**Mean ± SEM**	**Mean ± SEM**	***t***	**df**	***P *** **value**
OPRK1	8.59 ± 025	8.45 ± 0.14	<1.0	33	N.S.	7.17 ± 0.24	8.32 ± 0.25	3.28	23	**<0.005**^**a**^
PDYN	2.51^b^ ± 0.27	2.07^b^ ± 0.15	1.45	39	NS	8.05 ± 0.29	7.19 ± 0.13	3.06	37	**<0.005**
OPRM1	7.16 ± 0.16	7.07 ± 0.16	<1.0	35	N.S.	8.33 ± 0.18	8.44 ± 0.18	<1.0	23	N.S.
CD68	6.34 ± 0.14	6.99 ± 0.12	3.31	34	**<0.005**	6.65 ± 0.24	7.56 ± 0.12	3.65	**23**	**<0.002**
CD163	6.85 ± 0.23	8.05 ± 0.21	3.71	34	**<0.001**	6.14 ± 0.34	7.37 ± 0.28	2.79	**22**	**<0.02**

^a^Genes with significant expression differences between HIV- and HIV+ individuals are shown in bold. ^b^PDYN mRNA levels in the caudate were measured using RNase protection assay as pg PDYN mRNA/ microgram total RNA.

NS, no significant difference between HIV- and HIV+. Levels of mRNA were determined using RT-PCR, and expressed as natural logarithm of cDNA copy number normalized to copies of GAPDH cDNA copy number.

### Relationship of expression of opioid genes with macrophage markers

**Figure 3:**
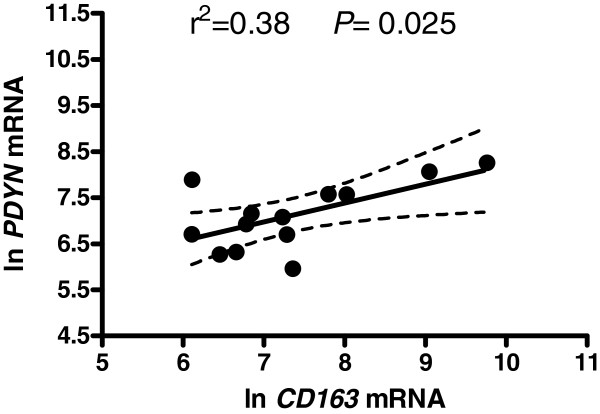
**There were positive correlations of *PDYN *** **and *OPRK1 *** **mRNA levels with the expression of the anti-inflammatory macrophage marker CD163 in the anterior cingulate of HIV infected subjects.**

An inflammatory response to HIV infection within the CNS is considered to be the major mediator of neuronal alterations in brain. Consistent with many other studies, we have found elevated mRNA levels of both proinflammatory CD68 and anti-inflammatory CD163 macrophage/microglial markers in both the caudate and anterior cingulate in HIV+ subjects (Table 1). In order to elucidate whether observed alterations in levels of opioid genes are associated with protective or detrimental processes in the HIV-infected brain, we performed linear regression analysis. There were significant positive correlations of *PDYN* and *OPRK1* mRNA levels (correlated with one another, see above) with the macrophage scavenger receptor CD163 in the anterior cingulate of HIV+ subjects (Figure 3). In contrast, within HIV+ subjects *OPRK1* and *PDYN* did not correlate with levels of the microglial marker CD68.

### Relationship to neuropsychological impairment

**Figure 4:**
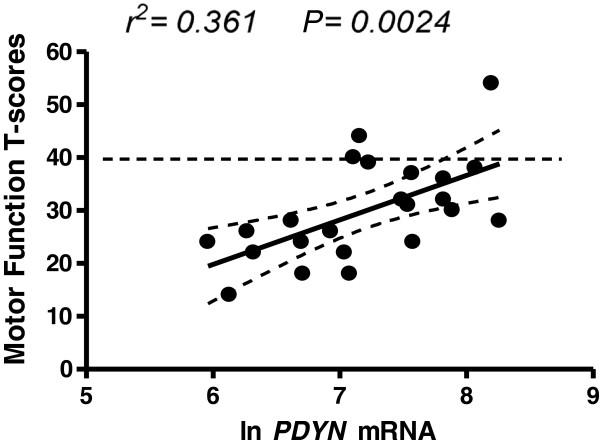
**There was a positive correlation of motor T-scores with *PDYN *** **mRNA levels in the anterior cingulate in HIV positive subjects, showing that higher levels of *PDYN *** **mRNA were associated with better neuropsychological test performance.**

To determine whether levels of *PDYN* and *OPRK1* mRNA were correlated with specific domain T-scores [see Additional file 1: Table S1], we performed linear regression analysis. Among the seven neurocognitive domains (see Methods), there was a positive correlation of motor T-scores with *PDYN* mRNA levels in the anterior cingulate in HIV-positive subjects (*P* <0.005, Figure 4). We did not find a correlation of any other neurocognitive domain T-scores with levels of *OPRK1* or *OPRM1* mRNA levels in this region.

Separate analyses showed no significant differences in scores of each neurocognitive domain in our samples between genders, or between HIV-positive subjects on an antiretroviral therapy (ART) and those not on ART; there was also no significant correlation of age with motor function domain scores in the HIV+ subjects.

## Discussion

The major findings of the present study are that:

1. There were opposite directions of change in the levels of *PDYN* and *OPRK1* mRNA in the anterior cingulate in postmortem brain of HIV+ subjects: lower levels of *PDYN* with greater levels of *OPRK1*.

2. There were positive correlations of *PDYN* and *OPRK1* levels with expression of the anti-inflammatory microglial/macrophage marker CD163. In contrast, within HIV+ subjects, *OPRK1* and *PDYN* were not correlated with levels of proinflammatory CD68.

3. There was a positive correlation between better T-scores in the motor domain scale and *PDYN* mRNA levels in the anterior cingulate.

To our knowledge this is the first report of region-specific alterations in expression of these two opioid system genes in postmortem brain of HIV-infected individuals. One interesting result of the present study is the finding of lower levels of *PDYN* mRNA in the anterior cingulate in HIV-infected subjects. Other studies have reported an increase or no change in *PDYN* expression in postmortem brain in subjects with schizophrenia and other psychiatric disorders [36].

The prodynorphin gene contains several calcium-responsive enhancer elements in its promoter region, including calcium/cAMP responsive element, phorbol ester-responsive element, and downstream regulatory element (DREAM) and is highly responsive to calcium levels ([37] for review). Although dynorphins preferentially bind KOR and are potent and efficacious KOR agonists, several studies in cell culture and in the rodent CNS suggest that dynorphin peptides may potentiate glutamatergic receptor function and neurotoxicity, possibly through non-KOR sites of action [38-40]. However, in human neurodegenerative diseases there is currently no direct evidence in support of this proposed alternative dynorphin-mediated mechanism of neurotoxicity.

Studies of a rodent model of viral encephalitis based on Borna disease virus (BDV) showed dynorphin depletion in the hippocampus due to depopulation of the granule layer and loss of competence of surviving granule cells to express dynorphin [41]. Lower *PDYN* mRNA levels and dynorphin peptides were observed in several experimental animal models of neuropathological conditions [42-44]. Cell culture studies showed that activation of the human macrophage cells U-937 with lipopolysaccharide (LPS) led to a decrease in *PDYN* mRNA levels through transcriptional inhibition of gene expression [45].

Recently, significantly lower levels of another opioid neuropeptide mRNA, preproenkephalin (*PENK*) were found in the dorsolateral prefrontal cortex in subjects with HIV encephalitis (HIVE) compared to seronegative controls [32]. The authors did not find differences in *PENK* mRNA levels in HIV-infected subjects with and without neurocognitive impairment, and concluded that the lower PENK levels were related neuropathologically to HIVE.

Mechanistic *in vitro* studies support the hypothesis that the release of numerous factors by activated macrophages, glial cells and astrocytes could be a cause of elevated levels of *OPRK1* mRNA in the anterior cingulate of HIV+ subjects observed in our study. For example, incubation of the murine macrophage cell line J774 with the proinflammatory cytokine IFN gamma for 24 h led to upregulation of *Oprk1* expression at both transcriptional and protein levels [46]. Functionality of KOR in macrophages was demonstrated by Dynorphin-A (1-17)-induced phosphorylation of ERK1/2. Also, in adjuvant-induced inflammation in rats, the proinflammatory cytokine interleukin-1 beta (IL-1 beta) induced upregulation of *Oprk1* in dorsal root ganglia [47].

Clinical and experimental central nervous system injuries elicit an inflammatory response that comprises mostly activated macrophages [48]. These cells exist in a state of dynamic equilibrium within the lesion microenvironment. Thus, depending on the inflammatory conditions in the lesion microenvironment, they may differentiate into proinflammatory cells that aggravate tissue injury, or anti-inflammatory cells that promote CNS repair [49]. In our study, there was elevation of proinflammatory CD68 and anti-inflammatory CD163 mRNA in both the caudate and anterior cingulate of HIV+ subjects. Also, levels of *PDYN* and *OPRK1* mRNA were positively correlated with CD163 mRNA in the anterior cingulate, but not in the caudate. In contrast, within HIV+ subjects mRNA levels of *OPRK1* and *PDYN* were not correlated with levels of CD68. In a rodent model of Parkinson’s disease, treatment with 1-methyl-4-phenyl-1,2,3,6-tetrahydropyridine (MPTP) or methamphetamine led to higher levels of proinflammatory macrophages (CD16, CD32 and CD86) in dynorphin knockout mice (Dyn^-/-^) than the wild-type, suggesting anti-inflammatory and neuroprotective properties of *PDYN* gene products (dynorphin peptides) [50]. MPTP-induced more severe motor deficits in Dyn-/- than in wild-types, and Dyn-/- mice also exhibited greater dopaminergic depletion. This suggests that endogenous dynorphins play an important role in protection of nigrostriatal DAergic neurons from chemical insults.

One of our findings is a positive correlation between levels of anterior cingulate *PDYN* mRNA levels and better T-scores in motor domain scale in HIV+ subjects. Animal models of experimental traumatic brain injury (TBI) provide some clues to a role of dynorphin and kappa opioid receptors in spatial memory and motor tasks. For example, TBI in rat resulted in increased *Pdyn* mRNA and dynorphin peptide levels in hippocampus, and intracerebroventricular administration of the KOR antagonist nor-BNI exacerbated motor and vestibulomotor deficits [19]. Of the cognitive domains we assessed, the association with motor performance likely reflects both a neurochemical association as well as a neuroanatomic specificity. The lack of association with other cognitive domains may be, in part, a function of the brain regions we examined and those we did not. For example, we did not assess the hippocampus, which is critical to learning and memory, nor did we examine orbitofrontal and dorsolateral prefrontal regions, implicated in executive functioning. On the other hand, the association of anterior cingulate with initiation of motor activity has been documented in humans, and is in keeping with the anatomic localization of our findings [51]. Thus, further study of other brain regions may be necessary to fully understand the extent of association between DYN/KOR and cognitive processes in humans.

The present study has several limitations that could be the focus of larger follow-up studies. The alterations in levels of *PDYN/OPRK1* system and macrophage markers in brain of HIV-infected subjects were measured only at the mRNA level, and not the peptide/protein gene products. Cell heterogeneity in the samples may also be a consideration, since *OPRK1* is expressed in diverse cell types and phenotypes (for example, neurons, microglia, macrophages). This may potentially ‘mask’ a more specific relationship of *OPRK1* or *PDYN* with HIV-associated neurocognitive impairment. Of interest, the anterior cingulate cortex (ACC) can be divided anatomically and functionally into distinct subregions, dorsal and ventral ACC. The dorsal ACC is connected with the prefrontal cortex, parietal cortex and the motor system. The ventral part of the ACC is connected with the amygdala, nucleus accumbens, hypothalamus, and anterior insula. Moreover, a study of the distribution of binding sites of 15 neurotransmitter receptors showed a differential pattern of expression of glutamate, GABA, acetylcholine, serotonin, and dopamine receptors among ACC subregions and neurons in humans [52]. It would be of great interest to study an interaction of the DYN/KOR system with other receptors in specific ACC regions. In addition to cell heterogeneity, postmortem tissues cannot be rigorously controlled for terminal events and certain medical factors; this variability may mask associations in relatively small ‘n’ study. Thus, our findings need replication in larger groups of individuals, both with and without HIV infection.

## Conclusions

In summary, this is the first report indicating alterations of dynorphin and kappa opioid receptor mRNA levels in the brain of HIV+ subjects. The decrease of *PDYN* mRNA levels in the anterior cingulate of HIV+ subjects compared to controls is related to inflammatory-mediated neuronal and dendritic loss. A positive correlation of better T-scores in motor domain scale with *PDYN* mRNA levels within HIV+ subjects may indicate that higher dynorphin expression is involved with protection from neurodegeneration and loss of function at sites of brain lesions. We also hypothesize that higher levels of *OPRK1* mRNA found in the anterior cingulate in HIV-infected subjects may represent a compensatory neuroprotective function of the PDYN/OPRK1 system in response to inflammation-induced excitotoxic neuronal damage. Based upon work in other models of neurodegeneration and neuroinflammation, the *PDYN*/*OPRK1* system has emerged as having neuroprotective properties and the ability to dampen pro-inflammatory responses of macrophages, lymphocytes, astrocytes and glial cells, properties that may have a positive influence in HIV-1 neuropathogenesis. Further studies are required for a better understanding of the role of KOR and their endogenous ligand dynorphin in HIV neuropathogenesis. These studies could lead to the development of novel pharmacotherapeutic approaches for neuroinflammatory/neurodegenerative disorders, including HAND, based on actions at kappa opioid receptors.

## Abbreviations

ACC: Anterior cingulate cortex; cART: Combination antiretroviral therapy; CD163: Scavenger receptor, monocyte/microglia/macrophage marker; CD68: Glycoprotein, monocyte/microglia/macrophage marker; Ct: Threshold cycles; DLFPC: Dorsolateral prefrontal cortex; DOR: Delta opioid receptor; GAPDH: Glyceraldehyde-3-phosphate dehydrogenase; HAND: HIV-associated neurocognitive disorder; HIVE: HIV encephalitis; KOR: Kappa opioid receptor; MOR: Mu opioid receptor; OPRK1: Kappa opioid receptor gene; OPRM1: Mu opioid receptor gene; PDYN: Prodynorphin peptide gene; PENK: Preproenkephalin; RIN: RNA integrity number; RT-PCR: Real-time polymerase chain reaction; TBI: Traumatic brain injury.

## Competing interests

The authors declare that they have no competing of interests.

## Authors’ contributions

VY performed extraction of RNA, quantification of mRNA levels using RT-PCR, and drafted the manuscript. SM provided postmortem brain material, dissected brain regions and was involved in data analyses and writing the manuscript. AH performed the statistical analyses. ERB was involved in writing and editing the manuscript. MJK participated in study design, interpretation of data and editing the manuscript. All authors read and approved the final manuscript.

## Supplementary Material

Additional file 1: Table S1 Study HIV-positive subjects’ information. Click here for file

Additional file 2: Table S2 Primer sequences for the quantitative RT-PCR SYBR Green assay. Click here for file
