# Supplementary material for: Neurocognitive and neuroinflammatory correlates of PDYN and OPRK1 mRNA expression in the anterior cingulate in postmortem brain of HIV-infected subjects
Source: J Neuroinflammation. 2014 Jan 9;11:5. doi: 10.1186/1742-2094-11-5 (PMC3896676; doi:10.1186/1742-2094-11-5)
Supplement: Additional file 1: Table S1 — Study HIV-positive subjects’ information. [file 1742-2094-11-5-S1.docx]

| **Supplement Table 1.** Study HIV positive subjects information | | | | | | | | |  |  |  |  |  |  |  |
| --- | --- | --- | --- | --- | --- | --- | --- | --- | --- | --- | --- | --- | --- | --- | --- |
|  |  |  |  |  |  |  |  |  |  |  |  |  |  |  |  |
|  | | | | | | | | T-scores | | | | | | | |
| PID | Category | Race | Age | Sex | PMI (hrs) | Plasma viral load | CD4 | Global | Motor | Information processing | Working memory | Memory encoding | Memory retrieve | Fluency | Executive functioning |
| 10001 | HIV | AA | 64 | F | 4.5 | 359 | 72 | 42 | 37 | 41 | 48 | 36 | 34 | 64 | 43 |
| 10002 | HIV | AA | 58 | M | 15.5 | <50 | 459 | 57 | 44 | 57 | 70 | 55 | 44 | 72 | 60 |
| 10003 | HIV | C | 45 | M | 8 | - | - | 35 | 24 | 52 | 56 | 19 | 10 | 42 | 34 |
| 10011 | HIV | H | 44 | M | 8.5 | 162642 | 16 | 44 | 40 | 43 | 32 | 50 | 50 | 51 | 48 |
| 10013 | HIV | C | 33 | M | 17 | >750000 | 9 | 49 | 24 | 59 | 63 | 48 | 46 | 52 | 50 |
| 10015 | HIV | C | 33 | M | 20 | 176800 | 66 | 35 | 18 | 48 | 60 | 28 | 26 | 47 | 28 |
| 10016 | HIV | AA | 58 | F | 17 | 576000 | 98 | 32 | 28 | 34 | 35 | 34 | 27 | 52 | 27 |
| 10023 | HIV | AA | 53 | M | 4.5 | 183 | 7 | 34 | 24 | 31 | 54 | 32 | 26 | 47 | 34 |
| 10025 | HIV | H | 46 | M | 7.5 | 73 | 93 | 28 | 22 | 29 | 31 | 24 | 20 | 42 | 36 |
| 10027 | HIV | H | 39 | M | 6 | 120714 | 109 | 27 | 39 | 25 | 26 | 18 | 20 | 33 | 36 |
| 10034 | HIV | H | 49 | M | 5 | 57712 | 77 | 28 | 18 | 29 | 32 | 22 | 26 | 37 | 38 |
| 10043 | HIV | AA | 34 | F | 17 | 2857 | 757 | 35 | 32 | 34 | 42 | 32 | 38 | 32 | 34 |
| 10045 | HIV | H | 31 | F | 9 | 744349 | 4 | 27 | 14 | 29 | 37 | 24 | 31 | 20 | 35 |
| 10052 | HIV | AA | 39 | M | 5 | 174175 | 79 | 40 | 26 | 40 | 58 | 38 | 36 | 61 | 32 |
| 10063 | HIV | H | 51 | M | 5 | 65 | 136 | 24 | 26 | 28 | 28 | 10 | 10 | 34 | 36 |
| 10064 | HIV | C | 49 | M | 12 | - | 278 | 27 | 20 | 27 | 33 | 22 | 21 | 34 | 38 |
| 10065 | HIV | H | 46 | M | 7 | >750000 | 18 | 41 | 38 | 50 | 50 | 26 | 30 | 33 | 49 |
| 10066 | HIV | AA | 54 | F | 7 | 469163 | 8 | 33 | 36 | 30 | 50 | 26 | 26 | 34 | 40 |
| 10074 | HIV | AA | 41 | M | 17.5 | >750000 | 3 | 31 | 28 | 41 | 38 | 22 | 16 | 35 | 34 |
| 10086 | HIV | AA | 48 | F | 7.5 | 22957 | 249 | 36 | 32 | 38 | 47 | 33 | 32 | 40 | 36 |
| 10094 | HIV | H | 41 | F | 6.5 | 45413 | 1 | 26 | 30 | 34 | na | 10 | 26 | na | 23 |
| 10103 | HIV | H | 40 | M | 6 | >750000 | 15 | 29 | 31 | 34 | na | 20 | 20 | na | 37 |
| 10133 | HIV | C | 48 | M | 7.5 | 173921 | 3 | 47 | 54 | 49 | 53 | 32 | 43 | 49 | 46 |
| 20024 | HIV | C | 62 | M | 4 | <50 | 20 | 25 | 22 | 25 | 27 | 17 | 18 | 38 | 33 |
| Abbreviatins: PID - subject I.D. at MHBB; | | | | |  |  |  |  |  |  |  |  |  |  |  |
| Race: AA-African American; C-Caucasians; H- Hispanics; A-Asians; PMI- post-mortem interval; | | | | | | | | | |  |  |  |  |  |  |
| T-scores calculated using normative data as described in the literature (Woods et al., 2004) | | | | | | | | |  |  |  |  |  |  |  |
