# Supplementary material for: Neurocognitive and neuroinflammatory correlates of PDYN and OPRK1 mRNA expression in the anterior cingulate in postmortem brain of HIV-infected subjects
Source: J Neuroinflammation. 2014 Jan 9;11:5. doi: 10.1186/1742-2094-11-5 (PMC3896676; doi:10.1186/1742-2094-11-5)
Supplement: Additional file 2: Table S2 — Primer sequences for the quantitative RT-PCR SYBR Green assay. [file 1742-2094-11-5-S2.docx]

| Supplement Table 2 | |  |  |  |  |
| --- | --- | --- | --- | --- | --- |
| Primer sequences for the quantitative RT-PCR SIBR Green assay | | | |  |  |
| Custom designed primers | |  |  |  |  |
| Genes | Gene | Forward (5' -> 3') | Reverse (5' -> 3') | Amplicon |  |
|  | reference |  |  | length, bp |  |
| OPRM1 | NM_000914 | CCCTGCCCTTCCAGAGTGT | TGGCAGACTGCAATGTATCGA | 154 |  |
| PDYN | NM_024411 | GGTGCTCCTTGTGTGCTGTAA | GCATCTCTCCCATTCCTCAGA | 113 |  |
| CD163 | NM_004244 | ACCTGCTGTCTGGCAATGTA | GCTGCCTCCACCTCTAAGTC | 124 |  |
|  |  |  |  |  |  |
| Commercial primers (SABiosciences, www.SABiosciences.com) | | |  |  |  |
| OPRK1 (Cat. # PPH01883E) | |  |  |  |  |
| CD68 (Cat. # PPH05574F) | |  |  |  |  |
|  | |  |  |  |  |
|  | |  | | | |
|  | |  |  |  |  |
|  | | |  |  |  |
|  | |  |  |  |  |
|  | |  |  |  |  |
